# Polycomb Group Proteins Bind an *engrailed* PRE in Both the “ON” and “OFF” Transcriptional States of *engrailed*

**DOI:** 10.1371/journal.pone.0048765

**Published:** 2012-11-06

**Authors:** Kristofor K. Langlais, J. Lesley Brown, Judith A. Kassis

**Affiliations:** Program in Genomics of Differentiation, *Eunice Kennedy Shriver* National Institute of Child Health and Human Development, National Institutes of Health, Bethesda, Maryland, United States of America; St. Georges University of London, United Kingdom

## Abstract

Polycomb group (PcG) and trithorax Group (trxG) proteins maintain the “OFF” and “ON” transcriptional states of HOX genes and other targets by modulation of chromatin structure. In Drosophila, PcG proteins are bound to DNA fragments called Polycomb group response elements (PREs). The prevalent model holds that PcG proteins bind PREs only in cells where the target gene is “OFF”. Another model posits that transcription through PREs disrupts associated PcG complexes, contributing to the establishment of the “ON” transcriptional state. We tested these two models at the PcG target gene *engrailed*. *engrailed* exists in a gene complex with *invected*, which together have 4 well-characterized PREs. Our data show that these PREs are not transcribed in embryos or larvae. We also examined whether PcG proteins are bound to an *engrailed* PRE in cells where *engrailed* is transcribed. By FLAG-tagging PcG proteins and expressing them specifically where *engrailed* is “ON” or “OFF”, we determined that components of three major PcG protein complexes are present at an *engrailed* PRE in both the “ON” and “OFF” transcriptional states in larval tissues. These results show that PcG binding per se does not determine the transcriptional state of *engrailed*.

## Introduction

The Polycomb group (PcG) and trithorax group (trxG) proteins are key regulators of genomic programming and differentiation in multicellular organisms [1]–[3]. In *Drosophila*, PcG proteins are present in at least 5 distinct multiprotein complexes, Pho Repressive Complex (PhoRC), Polycomb Repressive Complex 1 (PRC1), Polycomb Repressive Complex (PRC2), Polycomb repressive deubiquitinase (PR-DUB), and d-Ring-associated factors complex (dRAF) [4]–[6]. These complexes repress target gene expression through post-translational covalent modification of histones and modulation of chromatin structure. PhoRC consists of dSfmbt and the DNA-binding protein Pleiohomeotic (Pho). PRC1 is composed of Posterior Sex Combs (Psc), Polyhomeotic (Ph), Polycomb (Pc), and the H2A K119 ubiquitylase dRing/Sce. dRAF consists of dRing/Sce, Psc, and dKdm2 [5]. PRC2 contains Extra Sex Combs (Esc), p55, Supressor of Zeste 12 (Su(z)12), and Enhancer of Zeste (E(z)), which is responsible for placing the H3K27me3 mark thought to indicate repressive chromatin. In *Drosophila*, PcG protein complexes are targeted to specific genomic sites by DNA regions called Polycomb group Response Elements (PREs) [7], [8].

The presence of PcG proteins and H3K27me3 at a target gene usually indicates a repressed transcriptional state [9]. However, many studies suggest this is not always the case. Notably, many developmentally important genes are associated with both H3K27me3 and H3K4me3 (the active chromatin mark) in embryonic stems cell, the so-called “bivalent state,” and are transcribed at a low level [10], [11]. However, a recent study showed that the “bivalent state” for the genes tested did not exist, but was only an indication of a mixed cell population [12]. In Drosophila, a few studies have shown PcG protein binding to transcribed genes. In Drosophila imaginal disk cells, Papp and Müller found PcG proteins bound to *Ubx* PREs in both wing disks, where its transcription is off, and in the leg and haltere disks, where *Ubx* is transcribed [13]. PREs of the ubiquitously-expressed Psc gene are also bound by PcG proteins in imaginal disk cells [14]. Further, genome-wide studies comparing PcG target genes in three different tissue culture cell lines suggest the presence of at least 4 PcG states [15], fully repressed (with just PcG proteins bound to the PRE), fully active (with just trxG proteins bound to the PRE), ‘balanced’ (with PcG and trxG proteins bound to the PRE), and void (with neither PcG nor trxG proteins bound to the PRE). Of particular interest for this study, the *engrailed* (*en*) and *invected* (*inv*) genes exist in a fully repressed state in Sg4 cells (a line originally derived from late embryos), but are in a balanced state, with trxG and PcG proteins bound to the PREs, and H3K27me3 extending over the two transcription units in BG3 cells (a line derived from neuronal tissue) where they are also bound by RNA Polymerase II and are transcribed [15], [16]. These results indicate that at *en* and *inv*, at least in BG3 cells, transcription and PcG protein binding are not mutually exclusive.

It has been proposed that transcription through PREs antagonizes PcG protein complex activity and plays a key role in setting up the “ON” transcriptional state [17]–[21]. At the Bithorax complex (BC-X), which includes the genes *Ubx*, *Abd-A*, and *Abd-B*, there are at least a dozen ncRNAs transcribed in embryos [22]. Numerous studies show that transcription through PREs of the BC-X can interfere with maintenance of PcG-mediated silencing [17]–[19]. In reporter gene experiments, transcription through a PRE was not only shown to inactivate it, but to change its activity to a transcriptional activator instead of a silencer [20]. At the *en* gene, it was reported that the *en* PRE was transcribed in embryos, but not in larvae, suggesting that *en* PRE activity could be regulated by different mechanisms in different developmental stages [20].

The PcG targets *en* and *inv* are adjoining, co-regulated genes, that share regulatory DNA [23]. There are four major *en/inv* PREs, two upstream of *inv* and two closely spaced PREs just upstream of the *en* transcription unit [24], [25]. The two well-characterized *en* PREs are within 1 kb of each other and often appear as a single binding peak for PcG proteins in chromatin immunoprecipitation experiments. *en* and *inv* PREs are bound by PcG proteins in tissue culture cells, embryos, larvae, and adults [26]–[28]. Further, *inv* and *en* comprise a H3K27me3 domain that covers a 115kb region, ending abruptly at the 3′ ends of the *Enhancer of Polycomb* (*E(Pc)*) and *toutatis* (*tou*), the transcription units that flank the region [29]. We used in situ hybridization to embryos to examine how much of the *en/inv* domain is transcribed and in what pattern. Unlike the BX-C with its abundant ncRNA, ncRNAs are relatively rare in the *en/inv* domain. Further, we found no evidence for transcription of the *inv* or *en* PREs. Genome-wide PcG-binding studies in embryos, larvae, and adults show the locations of PcG binding to *en* in mixed cell populations [26]–[28]. However, it was not known whether PcG proteins are bound to the PRE in vivo in cells where *en* is expressed. In order to examine this, we expressed FLAG-tagged PcG proteins specifically in cells where En is “ON” or “OFF”, and used chromatin immunoprecipitation with FLAG antibodies to determine FLAG-PcG protein binding to the *en* PRE. Our results show that PcG proteins are bound to the *en* PRE both in cells that express *en* and those that don't. This shows that PcG binding per se is not sufficient to silence *en/inv* expression.

## Results

### Analysis of ncRNAs in the *en-inv* region

**Figure 1:**
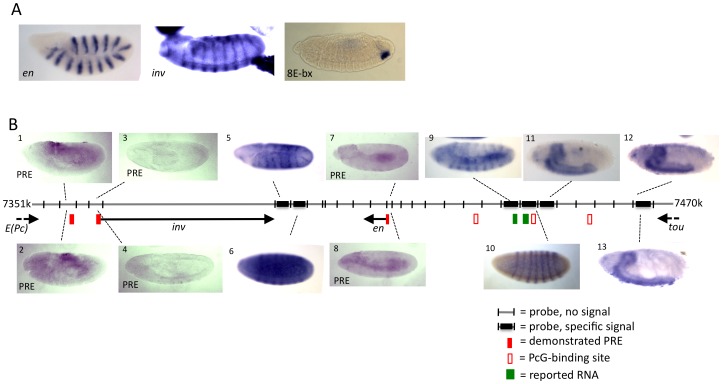
Whole mount embryo in situ hybridization reveals that ncRNAs are not detectable at the known *en* and *inv* PREs. Grey Line indicates genomic DNA, with the coordinates listed at both ends (genome version R5.1). DIG-labeled RNA probes were generated to cover the entire region shown, on both strands. (A) Positive controls showing robust signal from *en* and *inv* probes, and from a probe against *miR-iab-8*, a miRNA in the BX-C [30]. (B) Selected in situ results from *inv-en* region. Panels 1–4 and 7, 8 show non-specific background staining using probes to detect RNAs transcribed in the regions of the *inv* and *en* PREs. Several probes yielded specific signals. Panels 5 and 9 show an *en*-like pattern at stage 9, panels 6 and 10 show a pair-rule pattern at stage 5, and panels 11–13 show late CNS staining at stage 16. Embryos located above the genomic DNA line were hybridized with antisense probes (with respect to *inv*), embryos located below the line were hybridized with sense probes (with respect to *inv*). Filled red boxes are the locations of PREs (as evidence by PcG binding and by PRE activity in transgenes). PcG protein binding sites, depicted with open red box, are where Pho was reported to bind in ChIP/chip studies in larvae and embryos [39]. Green boxes indicate the locations of regions reported to be transcribed [31], [32].

*inv* and *en* comprise a 115 kb domain flanked by the 3′ end of the genes *E(Pc)* and *tou* (Fig. 1). We conducted *in situ* RNA hybridization on whole embryos, using DIG-labeled RNA probes designed to recognize RNAs transcribed in either direction throughout the entire 115 kilobase domain (Fig. 1). Positive control probes were made against the *en* and *inv* transcripts, and against a nc RNA encoding a micro-RNA arising from the *iab-8* region in the BX-C. This probe yielded a robust signal in the A8 region (Fig. 1), as described previously [30]. No specific signal was detected within the interval between the 3′ end of *E(Pc)* and the 5′ end of *inv* region, which contains two *inv* PREs (Figure 1B, panels 1–4). In the *inv-en* intergenic region, a specific signal resembling the *inv* expression pattern (Fig. 1A) was obtained using a probe just downstream of the *inv* transcript (Fig. 1B, panel 5). We suspect that this signal could be the result of transcriptional read through. In the next fragment, a transient pair-rule expression pattern was detected using a probe from the other strand (Fig. 1B, panel 6). Moving to the region upstream of the *en* transcription unit, no specific signal was observed with probes designed to detect transcription from the *en* PRE (Fig. 1B, panels 7 and 8). This result differs from what was reported by Schmitt et al. [20], who detected a weak stripe signal in germ band elongated embryos with a probe to the *en* PRE. We were also unable to detect this weak stripe signal using the exact probes used in their experiments (data not shown). Further upstream of the *en* transcript, probes yielded an *en*-like expression pattern (Fig. 1B, panel 9), and a pair-rule pattern (panel 10), in regions that contain previous experimental evidence of transcripts and a pair-rule enhancer [31], [32] (JAK unpublished data). Finally, still further upstream, central nervous system staining was observed in stage 17 embryos (panels 11, 12, and 13). The expression from probe 13 could be transcriptional read through from the *tou* gene.

We also examined polyA and non-polyA RNA-seq data from the ModEncode project [29]. No RNAs of either type were observed at any embryonic (0–24 hours) or larval stage in the *inv-en* or *en-tou* regions. However, a robust signal spanning 1100 bp (2R:7360200..7361299) was observed upstream of the *inv* promoter and adjacent to one of the two known *inv* PREs (PRE coordinates 2R:7362423..7363955 [24]) (Fig. 1B). This signal was observed in all stages, beginning in 0–1 hour embryos. This signal is likely an artifact however, as this 1100 bp region shows near sequence identity to 21 other regions in the genome. Taken together, these results suggest that ncRNAs are not as abundant in the *en/inv* region as they are in the BX-C, and that *inv* and *en* PREs are not transcribed in embryos. We also examined whether the *inv* and *en* PREs are transcribed in imaginal discs and the larval CNS and saw no evidence of transcription (data not shown). We note that Schmitt et al. also found no evidence of *en* PRE transcription in larval tissues [20].

### PcG proteins bind to the *en* PRE in both the “ON” and “OFF” transcriptional states of *en*

PcG protein binding to *en* and *inv* PREs has been examined in genome wide studies using embryos, larvae, and adults [26]–[28]. The samples in these studies contain a mixture of cells, some of which transcribe *en* and *inv*, and others that do not. *en* and *inv* exist in a “balanced” state in BG3 cells, with transcription in the presence of PcG binding [15], [16]. We wished to determine whether this was also the case in vivo. We used a UAS-driven FLAG-tagged PcG crosslinked-ChIP (X-ChIP) system to examine PcG binding in cells that express *en* and those that do not. *en* is expressed in stripes in embryos and in the posterior compartments of imaginal discs. *cubitus interruptus* (*ci*), is expressed in a complementary pattern with *en*, with no overlap in both embryos and imaginal discs [33]. By expressing UAS-FLAG-tagged proteins in specific cell populations with *en*-GAL4 and *ci*-GAL4 driver lines [34], it is possible to use ChIP to examine the binding profile of any PcG protein in the “ON” or “OFF” transcriptional states of *en*.

Fly lines with 3XFLAG-tagged Pho, dRing/Sce, Esc, and Scm were generated. These proteins were chosen because they are present in different PcG protein complexes and might preferentially bind in the “OFF” versus the “ON” transcriptional state. All proteins were first tagged at the C-terminus. C-terminally tagged Scm-FLAG acted in a dominant negative fashion when ubiquitously expressed in a wild-type background, as indicated by strong PcG-type transformations (data not shown). Therefore, we generated and proceeded with an N-terminally tagged FLAG-Scm protein, which did not produce a phenotype when expressed ubiquitously in a wild type background.

**Figure 2:**
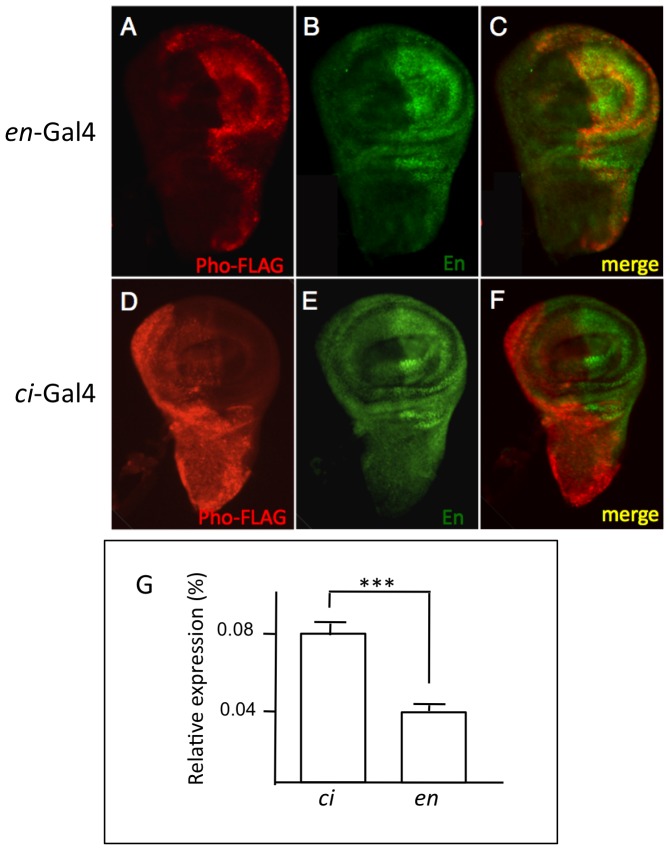
Stable expression of Pho-FLAG in cells that express En or Ci. UAS-Pho-FLAG expression by the *en*-GAL4 or *ci* -GAL4 driver. Anti-FLAG staining is red, anti-En staining is green. (A–C) 3^rd^ instar wing imaginal disc collected from a UAS-Pho-FLAG, *en*-GAL4 cross. Panel C shows nearly complete overlap of anti-FLAG and anti-En staining. (D–F) 3^rd^ instar wing imaginal disc collected from a UAS-Pho-FLAG, *ci*-GAL4 cross. Panel F shows complementary staining of anti-FLAG and anti-En. Note that the size of the anterior compartment, where Ci is expressed is about twice the size of the posterior compartment, where En is expressed [35]. (G) qRT-PCR showing that there is about twice as much Pho-FLAG transcript when it is driven by *ci*-Gal4 than by *en*-Gal4 (*** P≤0.001).

UAS-Pho-FLAG was crossed with *en*-GAL4 or *ci*-GAL4, and FLAG-expression was examined in whole embryos and imaginal discs from wandering 3rd instar larvae. As expected, Pho-FLAG driven by *en*-GAL4 was expressed in embryos (not shown) and in discs in a pattern that almost completely overlapped with endogenous *en* (Fig. 2A–C). Pho-FLAG driven by *ci*-GAL4 was expressed in a non-overlapping pattern complementary to endogenous *en* (Fig. 2D–F), consistent with the reported expression pattern of *ci*. Pho-FLAG expression was detected in a few cell of the CNS, coincident with cells that express En, when driven by the *en*-GAL4 driver (data not shown). There was no expression of Pho-FLAG in the CNS when driven by the *ci*-GAL4 driver (data not shown). These results confirm that FLAG-tagged proteins are expressed in the desired cell populations. Note that the posterior compartment comprises only about a third of the cells of the imaginal disc [35], thus there are about twice as many cells expressing FLAG-tagged proteins with the *ci*-driver as with the *en*-driver. Consistent with this, quantitative RT-PCR showed there is approximately twice as much Pho-FLAG mRNA in *ci-*driven samples versus *en*-driven samples (Fig. 2G).

**Figure 3:**
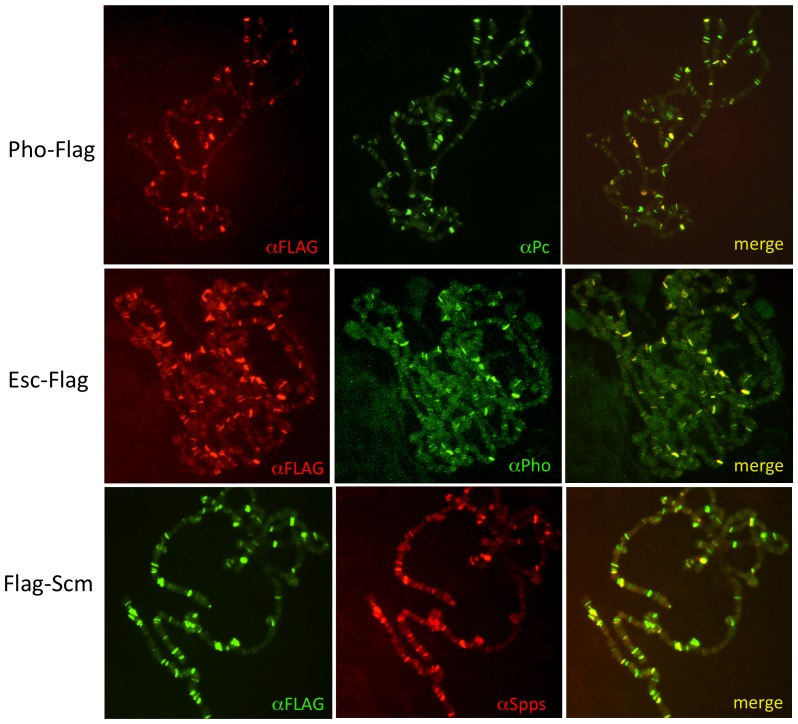
FLAG-tagged PcG proteins co-localize with endogenous PcG proteins on polytene chromosomes. FLAG-tagged proteins were driven by *arm*-Gal4.

Next, we compared the polytene chromosome-binding pattern of the FLAG-tagged proteins to the binding pattern of an endogenous PcG protein. For these experiments, FLAG-tagged proteins were driven ubiquitously with *arm*-GAL4. Pho-FLAG was detected on chromosomes in a pattern that completely overlapped with endogenous Polycomb (Pc) protein (Fig. 3A). There were some Pc bands that did not contain Pho-FLAG. There are two reasons for this: one, the detection of the Pho-Flag is relatively weak, and two, endogenous Pho does not bind all Pc sites in polytene chromosomes. Similarly, Esc-FLAG and Sce-FLAG largely overlap with endogenous Pho bands on polytene chromosomes (Fig. 3B and data not shown). For Scm, we examined the overlap with the PRE DNA binding protein Spps [36] and again saw a nearly complete overlap (Fig. 3C).

To test whether the FLAG-tagged proteins are functional, we ubiquitously expressed FLAG-tagged PcG proteins in flies with mutations or deletions for the respective genes to look for rescue. Esc-FLAG and Sce-FLAG completely rescued *esc* and *Sce* mutant flies, with no observable PcG or homeotic phenotypes. Pho-FLAG rescued *pho* flies with 10% of adult males showing moderate A4–A5 transformations. FLAG-Scm rescued *Scm* mutant flies, with about 70% of males exhibiting extra sex combs on the 2nd and 3rd legs. It is not surprising that minor PcG phenotypes are observed in some experiments, as the timing and level of expression of FLAG-tagged proteins, under the control of the UAS/GAL4 system, are not likely to perfectly match endogenous expression. Considering this, we conclude that the FLAG-tagged PcG proteins are functional, and that ChIP experiments carried out with these proteins would faithfully reflect results obtained with endogenous proteins.

**Figure 4:**
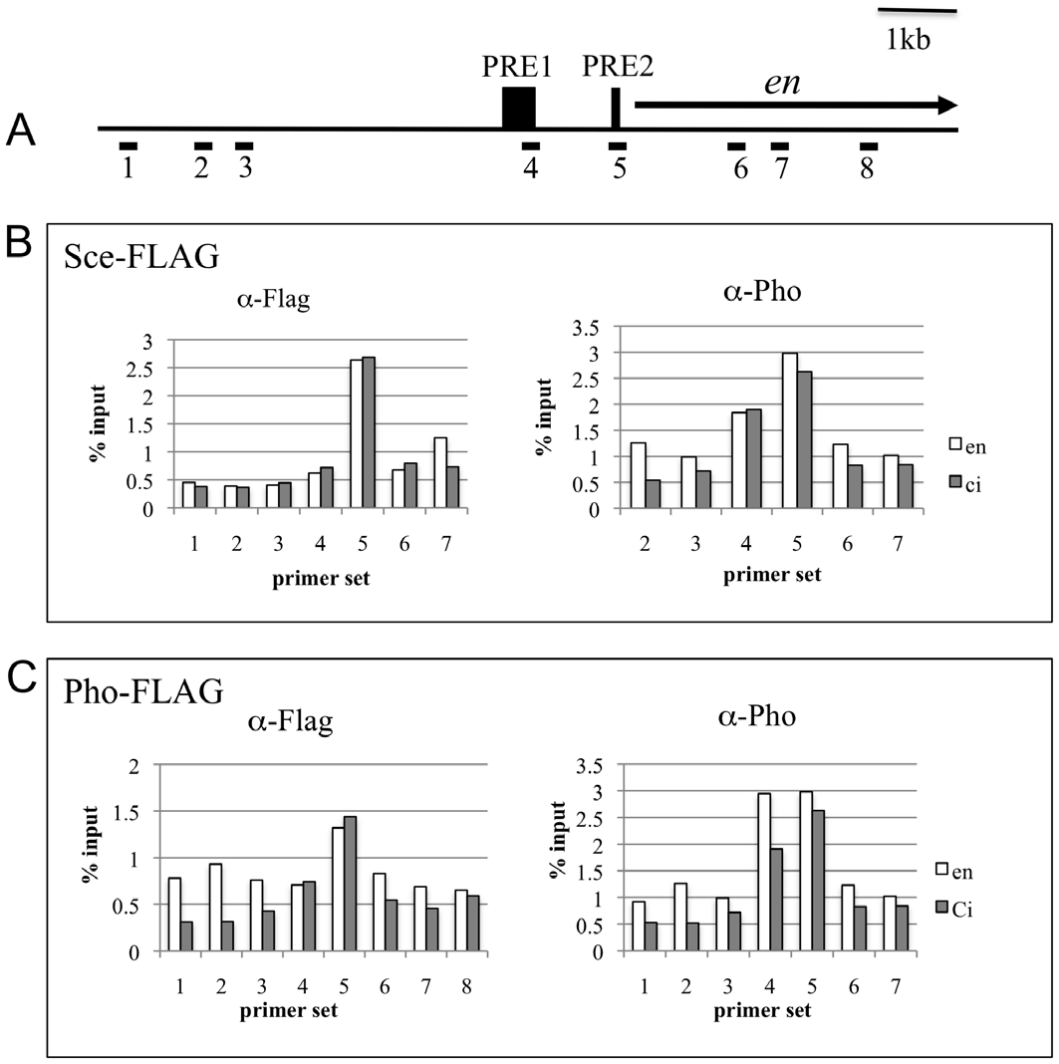
Pho-FLAG and Sce-FLAG binding peaks at PRE2. (A) A map of the *en* gene showing the location of the PREs and the probes used for the qPCR (#1–8). (B,C) Results of X-ChIP experiment with Sce-FLAG (B) and Pho-FLAG (C) driven by *en*-Gal4 (open bars) or *ci*-Gal4 (closed bars). Pho binding was also done on all chromatin preparations. The results of a representative experiment are shown. These experiments were done with a different batch of FLAG antibody and different ChIP reagents than those done in Fig. 5. Further, 20 larvae were used for each sample instead of 10. Under these conditions, we did not see a difference in the level of binding to the PREs between the “ON” and the “OFF” states; however, the qualitative result, PcG proteins binding to PRE2 in both the “ON” and “OFF” states was the same in these experiments and those in Fig. 5.

The validated FLAG-tagged proteins were used in X-ChIP experiments. FLAG-tagged PcG proteins were driven in flies with the *en*-GAL4 (“ON”) and *ci*-GAL4 drivers (“OFF”). Imaginal disc sets, along with the central nervous system, were collected from 3rd instar larvae, processed for X-ChIP, and analyzed with qPCR to determine binding signals at the *en* gene. The locations of the two PREs just upstream of *en* have been well characterized in functional studies (25–28; JLB and JAK, unpublished data) and are shown in Fig. 4A along with the *en* transcription unit and primer locations. The ChIP experiments were all done in flies that were wild type for all PcG genes, since these proteins must be expressed in all cells for proper development. *ci*- and *en*-driven Pho-FLAG and Sce-FLAG binding were measured using probes upstream and within the *en* transcription unit (Fig. 4). Sce-FLAG was bound to PRE2 in both the “ON” and “OFF” transcriptional states. Pho-FLAG has a similar binding profile except that binding to the non-PRE probes in the “ON” chromatin was higher than the “OFF” chromatin, and there was some binding to PRE1. For comparison, Pho binding was measured using the same chromatin used for the FLAG-samples. Pho ChIP measures binding in both the “ON” and the “OFF” cells. Note that the Pho-binding was similar in both the Pho-FLAG samples and the Sce-FLAG samples, suggesting that the Pho-FLAG accurately reflects the distribution of endogenous Pho.

**Figure 5:**
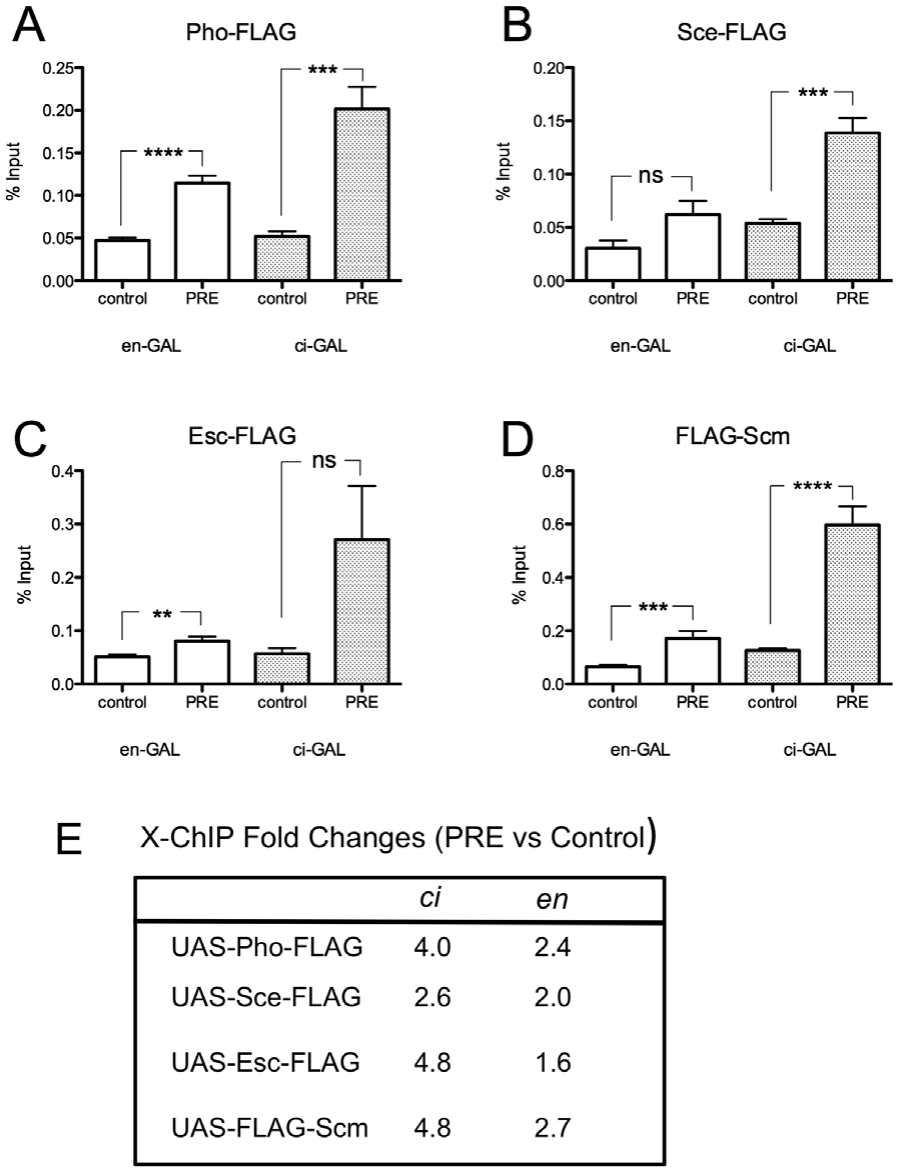
FLAG-tagged PcG proteins are bound to the *en* PRE in both the “ON” and “OFF” transcriptional states. (A–D) X-ChIP (with α-FLAG) was performed on third instar imaginal discs and CNS, with *en*-GAL4 or *ci*-GAL4 driven Pho-FLAG (A), Sce-FLAG (B), Esc-FLAG (C), FLAG-Scm (D). Results are shown as a percentage of the input DNA, collected prior to ChIP. ns = not significant, * P≤0.05, ** P≤0.01, *** P≤0.001, **** P≤0.0001 (un-paired, two-tailed t-tests). Results shown are from three independent biological samples with 2 replicates each. (E) Fold increase (PRE/control) using the means from the experiments shown in A–D. The UAS-lines are shown on the left, with the drivers *en*-Gal4 (*en*) and ci-Gal4 (*ci*) on top.

We compared the level of X-ChIP binding to *en* PRE 2 with that of a control fragment from the *en* intron (probe 8) for all of the FLAG-tagged PcG proteins. Each experiment was repeated 3 times and the results were pooled in Fig. 5. Pho-FLAG, FLAG-Scm, Sce-FLAG, Esc-FLAG, were present at *en* PRE2 in both the “ON” and “OFF” transcriptional states of *en*. These ChIP results suggest that PcG proteins are present in the *en* “OFF” transcriptional state at higher levels than in the “ON” state. For example, the Pho-FLAG signal is 4 fold higher than the control signal in *en* “OFF” cells, compared with 2.4 fold in *en* “ON” cells (Fig. 5E). Similar results are observed with FLAG-Scm (4.8 vs. 2.7), Esc-FLAG (4.8 vs. 1.6), and less so with Sce-FLAG (2.6 vs. 2.0). However, it is important to note that there are more *ci*-cells than *en*-cells, so we cannot conclude from this data that the levels of PcG binding in the “OFF” state are higher than those in the “ON” state.

## Discussion

In this study we sought to learn more about PcG protein complex-mediated regulation of *en* expression, focusing on mechanisms operating through *en* PREs. First we investigated whether the *en* and *inv* PREs are transcribed, and found no evidence of transcription of the PREs either by *in situ* hybridization or by analysis of RNAseq data from the region. We conclude that transcription of *inv* or *en* PREs does not play a role in regulation of *en/inv* by PcG proteins. Second, using FLAG-tagged PcG proteins expressed in either *en* or *ci* cells, we found that PcG proteins are bound to the *en* PRE2 in both the “ON” and “OFF” transcriptional state in imaginal disks. Our data suggest that PcG protein binding to PRE2 is constitutive at the *en* gene in imaginal disks and that PcG repressive activity must be suppressed or bypassed in the cells that express *en*.

Transcription through a PRE in a transgene has been shown to inactivate it, and, in the case of the *Fab7*, *bxd*, and *hedgehog* PREs turn them into Trithorax-response elements, where they maintain the active chromatin state [19], [20], [37]. However, is this how PREs work in vivo? Available data suggest that this could be the case for the *iab7* PRE [17]–[19]. Transcription through the PREs of a few non-HOX PcG target genes, including the *en, salm*, and *till* PREs has been shown by in situ hybridization to embryos [20]. However, in contrast to the robust *salm* and *till* staining, the picture of *en* stripes using the *en* PRE probe was very weak and corresponded to a stage where transient invaginations occur that could give the appearance of stripes [20]. Further, there was no hybridization of the *en* PRE probe to regions of the head [20], where *en* is also transcribed at this stage. Our in situ hybridization experiments with probes to detect transcription of the *inv* or *en* PREs did not yield specific staining at any embryonic stage, or in imaginal discs. This finding is confirmed by absence of polyA and non-poly RNA signals in this region at any embryonic or larval stage, upon review of RNA-seq data from ModEncode [29].

Our results show that PcG proteins bind to *en* PRE2 even in cells where *en* is actively transcribed. In fact, one member of each of the three major PcG protein complexes, Pho from PhoRC, dRing/Sce from PRC1, and Esc from PRC2, as well as Scm, are constitutively bound to *en* PRE2 in all cells in imaginal discs. We note that dRing/Sce is also present in the PcG complex dRAF, which also includes Psc and the demethylase dKDM2 [5]. Further experiments would be necessary to see whether Sce-FLAG is bound to *en* DNA as part of the PRC1 complex, the dRAF complex, or both.

What are the differences between the “ON” and “OFF” transcriptional states? Our data suggest that there may be some differences in Pho binding to non-PRE fragments (Fig. 4). However, this data has to be interpreted with caution. The *en*-GAL4 driver is an enhancer trap in the *inv* intron [38] and contains an *en* fragment extending from −2.4 kb through the *en* promoter. Thus, it is possible that the *en*-GAL4 driver alters Pho binding in the *en/inv* domain. In fact, the increased Pho-binding to non-PRE probes in the “ON” versus the “OFF” state in the FLAG-Sce samples suggests that the presence of the *en*-GAL4 driver alters Pho binding slightly.

One unexpected result from these experiments was that FLAG-Sce binds to PRE2 but not to PRE1 (Fig. 4). This is an interesting result that needs to be followed up on. Recent ChIP-Seq data in our lab using imaginal disk/brain larval samples and the anti-Pho antibody show 5 additional Pho binding peaks between *en* and *tou*, which could be 5 additional PREs (S. De and JAK, unpublished data). Three of these correspond to Pho binding peaks already identified by Oktaba et al. [39]. ChIP-seq experiments with the FLAG-tagged proteins expressed in the “ON” and “OFF” transcriptional states would be necessary to ask whether the distribution of PcG-proteins is altered at any of the PREs or any other region of the *en/inv* domain.

In conclusion, our data allows us to rule out two simple models of PcG-regulation of the *en/inv* genes. First, the *en/inv* PREs are not transcribed, so this cannot determine their activity state. Second, PcG proteins bind to at least one of the PREs of the *en/inv* locus in the “ON” state, therefore a simple model of PcG-binding determining the activity state of *en/inv* is not correct. Perhaps the proteins that activate *en* expression modify the PcG-proteins or the 3D structure of the locus and interfere with PcG-silencing. While FLAG-tagged PcG proteins offer a good tool to study PcG-binding particularly in the “OFF” state, cell-sorting of *en* positive and negative cells will be necessary to study the 3D structure and chromatin modification of the *en/inv* locus.

## Materials and Methods

### RNA data analysis

The following ModEncode mRNA and ncRNA reads in *inv-en* genomic regions were examined: Small ncRNA read samples: GSM286604, GSM364902, GSM286613, GSE24540, GSM286605, GSM286606, GSM286607, GSM286611, E12_V082, E02_V081, YAF01_V084, YAM01_V083, GSM360256, GSM360257, GSM322208, GSM322245, GSM360260, GSM360262, GSM322219, GSM322338, YA_GSM280086, O_V063. RNA-seq mRNA read samples: 3358, 3317, E0.2884, E2.2885, E6.2887, E8.2888, E10.2889, E12.2890, E14.2891, E16.2892, E18.2893, E20.2894, E22.2895, L1.2872, L2.2873, L312.2874, L313.2875, L314.2876, L315.2877, P0.2878, P12.2879, P24.2880, P48.2881, P72.2882, P96.2883, YF1.2866, YF5.2868, YF30.2867, YM1.2869, YM5.2871, YM30.2870, Dm_SOLiD.

### Whole-mount *in situ* hybridization of embryos

Digoxigenin (DIG)-labeled RNA antisense probe synthesis and whole mount in situ hybridization was carried out as previously described [40], except that fragments ranging in size from 500 to 3500 bp were cloned from genomic DNA for use as templates for probe synthesis. Probes were not fragmented with carbonate buffer. Probe template primer sequences are located in Table S1.

### Construction of FLAG plasmids

FLAG-tagged PcG transformation constructs were generated with the Gateway Cloning System (Invitrogen, Carlsbad, CA). *Sce*, *esc*, *pho*, and *Scm* cDNA clones were obtained from the *Drosophila* Genomics Resource Center (BGDP Gold cDNAs: LD23953 (*Sce*), SD03549 (*esc*), RE17954 (*pho*), RE16782 (*Scm*)). To generate Gateway entry clones, cDNAs were amplified using Phusion High-Fidelity DNA Polymerase and cloned into pENTR/dTOPO (Invitrogen) (for primer sequences see Table S1). Destination vectors containing N-terminal or C-terminal 3XFLAG, pTFW and pTWF respectively, were obtained from Terence Murphy and are further described at http://www.ciwemb.edu/labs/murphy/Gateway%20vectors.html. Clone cassettes in pENTR/dTOPO were recombined into pTWF and pTFW with LR Clonase (Invitrogen) according to manufacturers instructions. The resulting constructs were fully sequenced and checked for mutations and recombination errors prior to use.

### Transgenic lines

UAS-PcG-FLAG transgenic lines were generated by injections into *w^1118^* embryos by Genetic Services (Sudbury, MA, USA).

### Chromosome Squashes

Squashes and immunofluorescent staining of polytene chromosomes were performed as described previously [41], using anti-Pc, anti-Pho, or anti-Spps (at 1∶100), and monoclonal mouse anti-FLAG M2 (Sigma, St. Louis, MO) at 1∶1000 dilution.

### Reverse Transcription-Quantitative Polymerase Chain Reaction

Imaginal discs, along with the central nervous system, mouth hooks, and some anterior cuticle were dissected from 3^rd^ instar larvae (5 per sample) from *ci*- and *en*-GAL4 driven Pho-FLAG larvae and immediately placed in PBS on ice. Total RNA was collected from the resulting samples using Trizol (Invitrogen) according to the manufacturers instructions. One-step RT-qPCR was performed with the QuantiTect SYBR Green RT-PCR Kit on a Roche Lightcycler 480 according to manufacturer instructions. Relative expression levels of Pho-FLAG transcript was calculated using the ΔC(T) method, and expressed as a percentage of RP49 expression level. Pho-FLAG primers amplify a fragment containing the 3′ end of *pho* gene and a portion of the FLAG-encoding sequence. Pho-FLAG primers: 5′-CCGTTTGTGGTATATGCAGA-3′, 5′-CGTCATGGTCTTTGTAGTC-3′. RP49 primers: 5′-CGGATCGATATGCTAAGCTGT-3′, 5′-CGACGCACTCTGTTGTCG-3′

### Rescue Crosses

FLAG-tagged constructs were driven ubiquitously with an Arm-GAL4 driver in the following mutant backgrounds: *Scm* ^K3^/*Scm* ^K4^ (unpublished pharate adult lethal alleles from James A. Kennison), *p^p^ mcp Sce^1^/Df(3R)BSC499*, *esc* ^21^ *b cn*/*esc^M20^* (*esc^M20^* is an unpublished *esc* allele obtained from Mark Mortin and James A. Kennison), *pho* ^1^/*pho* ^1^, using standard crossing schemes.

### Cross-linked Chromatin Immunoprecipitation (X-ChIP)

Imaginal discs, along with the central nervous system, mouth hooks, and some anterior cuticle were dissected from 3^rd^ instar larvae (10 larvae per sample) and immediately placed in Schneider's medium (Invitrogen) on ice. Disc sets were fixed in 2% formaldehyde (Ted Pella Inc, Redding, CA) fixing solution (50 mM Hepes pH 7.6, 100 mM NaCl, 0.1 mM EDTA, 0.5 mM EGTA) for 15 minutes, then rinsed in stop solution (PBS, 0.01% Triton X-100, 0.125 M Glycine) for 10 minutes, followed by 2×10 minute washes with wash solution (50vmM Tris, 10 mM EDTA, 0.5 mM EGTA, 0.25% Triton X-100). Fixed and washed samples were stored at −80°C in storage solution (10 mM Tris-HCl pH 8.0, 1 mM EDTA, 0.5 mM EGTA). Whole discs were placed in 300 µl of action buffer with Complete Protease Inhibitor Cocktail in a 1.5 microcentrifuge tube, and sonicated in BioRuptor UCD-300 (Diagenode, Denville, NJ) for 30 seconds on/30 seconds off for 20 cycles, high power, resulting in chromatin fragments tightly concentrating at 200 base pairs, with a diminishing smear up to 1500 base pairs. Remaining insoluble material was spun down at full speed for 1 min, and chromatin supernatant was transferred to a new tube. 10 µl of chromatin was removed (3.3% of total volume) and saved from each sample for input reactions. ChIP was performed with monoclonal mouse anti-FLAG M2 (Sigma) at 1∶700 dilution, and the Millipore Chromatin Immunoprecipitation Assay Kit (Millipore, Billerica, MA) and Protein G agarose/salmon sperm DNA (Millipore). ChIP and input samples were then placed in a 65°C heat block for 4 hours to reverse cross-links. All samples were then purified with standard phenol/chloroform extraction. DNA samples were ethanol precipitated overnight, washed with 75% ethanol, and resuspended in 100 µl of water.

### qPCR analysis of X-ChIP

ChIP samples were analyzed with qPCR using a Lightcycler 480 Real-Time PCR System (Roche Applied Science) and Lightcycler 480 DNA SYBR Green I Master Mix (Roche Applied Science). Primers are listed in Table S1.

## Supporting Information

Table S1 (XLS)
